# Evolution of morphology and microstructure of GaAs/GaSb nanowire heterostructures

**DOI:** 10.1186/s11671-015-0812-8

**Published:** 2015-03-01

**Authors:** Suixing Shi, Zhi Zhang, Zhenyu Lu, Haibo Shu, Pingping Chen, Ning Li, Jin Zou, Wei Lu

**Affiliations:** National Laboratory for Infrared Physics, Shanghai Institute of Technical Physics, Chinese Academy of Sciences, 500 Yu Tian Road, Shanghai, 200083 China; Materials Engineering, The University of Queensland, St. Lucia, Brisbane, QLD 4072 Australia; College of Optical and Electronic Technology, China Jiliang University, Hangzhou, China; Center for Microscopy and Microanalysis, The University of Queensland, St. Lucia, Brisbane, QLD 4072 Australia

**Keywords:** GaSb, Heterostructure nanowire, Core-shell, Wurtzite, Molecular beam epitaxy, 61.46.Km, 81.07.Gf, 68.37.Lp

## Abstract

In this paper, we successfully grow GaAs/GaSb core-shell heterostructure nanowires (NWs) by molecular beam epitaxy (MBE). The as-grown GaSb shell layer forms a wurtzite structure instead of the zinc blende structure that has been commonly reported. Meanwhile, a bulgy GaSb nanoplate also appears on top of GaAs/GaSb core-shell NWs and possesses a pure zinc blende phase. The growth mode for core-shell morphology and underlying mechanism for crystal phase selection of GaAs/GaSb nanowire heterostructures are discussed in detail.

## Background

In the past decades, intensive research interests have been devoted towards the study of III-V semiconductor nanowires (NWs) for their important role in the research of fundamental physical phenomena [1,2] and also great potentials in future device applications of nano-electronics, opto-electronics, thermoelectrics, etc. [3-5]. Furthermore, NW heterostructures, including axial and core-shell structures, can offer us more possibilities on the extent of sophistication of material systems, and this will also accelerate the realization of NW-related applications [6,7].

Previous literatures of antimonide-based NWs have proved their promises in infrared detectors, transistors, quantum computing devices, etc. [8-12]. More importantly, antimonide core-shell nanowires possess some attractive properties useful for nanoscale electronics. For example, the type II and type III band alignment of GaAs/GaSb and InAs/GaSb heterostructures can help to produce an accumulation of charge carriers on different sides of the core-shell interface, thus resulting in a high carrier concentration without intentional doping [13]. Moreover, transport path and charge carrier type of the core-shell NWs can be effectively tuned by adjusting the thickness of shell layer and applied bias voltage [7]. Meanwhile, core-shell nanowire structures were also applied to improve the light emission efficiency of core NWs by means of suppressing surface states [14,15]. Unfortunately, studies on antimonide-based core-shell nanowire heterostructures are still lacking [7,16].

Crystal structure is another main concern in III-V NWs, since structure polytypism [17-19] and planar defects (such as stacking defaults (SFs) and twins) have been popularly observed, which are unwanted for related device applications [20]. However, the well-controlled synthesis of III-V NWs with a pure wurtzite (WZ) or zinc blende (ZB) phase can also bring about new application possibilities, since different crystal structures can lead to different band gaps [21-23] and, in turn, different electronic and optical properties. In fact, some novel structures like WZ/ZB or ZB twinning superlattice have been proposed [24-27]. However, antimonide NWs with the ZB structure are dominating from the previous reports, and this keeps true even for ternary antimonide NWs with only a small Sb concentration [28]. Although Mandl et al. [19] and Pozuelo et al. [29] have reported WZ structure of antimonide NWs, there is still neither theoretical prediction nor experimental data about WZ-structured GaSb.

Here, we carry out a systematic investigation on MBE growth of GaAs/GaSb NW heterostructures. It is found that despite of the variation of V/III ratios, GaSb always forms a shell layer instead of an axial NW segment on GaAs NWs. Meanwhile, a top bulgy nanoplate of pure GaSb also often appears in as-grown GaAs/GaSb core-shell NWs. A detailed structural characterization indicates that both GaAs core and GaSb shell NWs possess a WZ crystal structure, but a pure ZB phase forms for pure GaSb bulgy nanoplate. The growth mechanism of GaAs/GaSb core-shell NWs has been clarified at last.

## Methods

A Riber 32 R@D MBE system (Paris, France) was used to grow GaAs/GaSb NW heterostructures on the GaAs (111)_B_ substrates with Au catalysts. First, a pretreatment procedure including degassing and deoxidization was executed for all epi-ready GaAs substrates, in order to remove any possible contaminant on the substrates. Then, a GaAs buffer layer was grown for all samples under a temperature of 550°C for 15 min. Later, all substrates underwent an Au deposition and annealing step, so as to form isolated Au nanoparticles. Next, NW growth began. For all samples, GaAs NWs were first grown for 15 min, and then GaSb growth followed. The same growth condition was employed to grow GaAs NWs. Namely, growth temperature, Ga beam equivalent pressure (BEP), and As BEP were set at 380°C, 2.6 × 10^−7^ Torr, and 4.0 × 10^−6^ Torr, respectively. Then, GaSb NWs were grown under four different V/III ratios, while keeping the growth temperature and Ga flux as constants. Specifically, GaSb growth temperature was also set at 380°C, Ga BEP was 2.0 × 10^−7^ Torr, and V/III BEP ratio was set at 0.8, 1.3, 2.1, and 6.5, respectively. The growth switch between GaAs and GaSb NW was conducted by directly turning on the Sb source and shutting down the As source simultaneously. Once NW growth ended, both Ga source and Sb source shutters were closed immediately.

Detailed morphological and structural characterizations were performed on as-grown GaAs/GaSb heterostructure NWs through scanning electron microscopy (SEM; JEOL 7800 F, JEOL Ltd., Akishima, Tokyo, Japan) and transmission electron microscopy (TEM; Phillips Tecnai F20, Hillsboro, OR, USA, operated at 200 kV).

## Results

**Figure 1:**
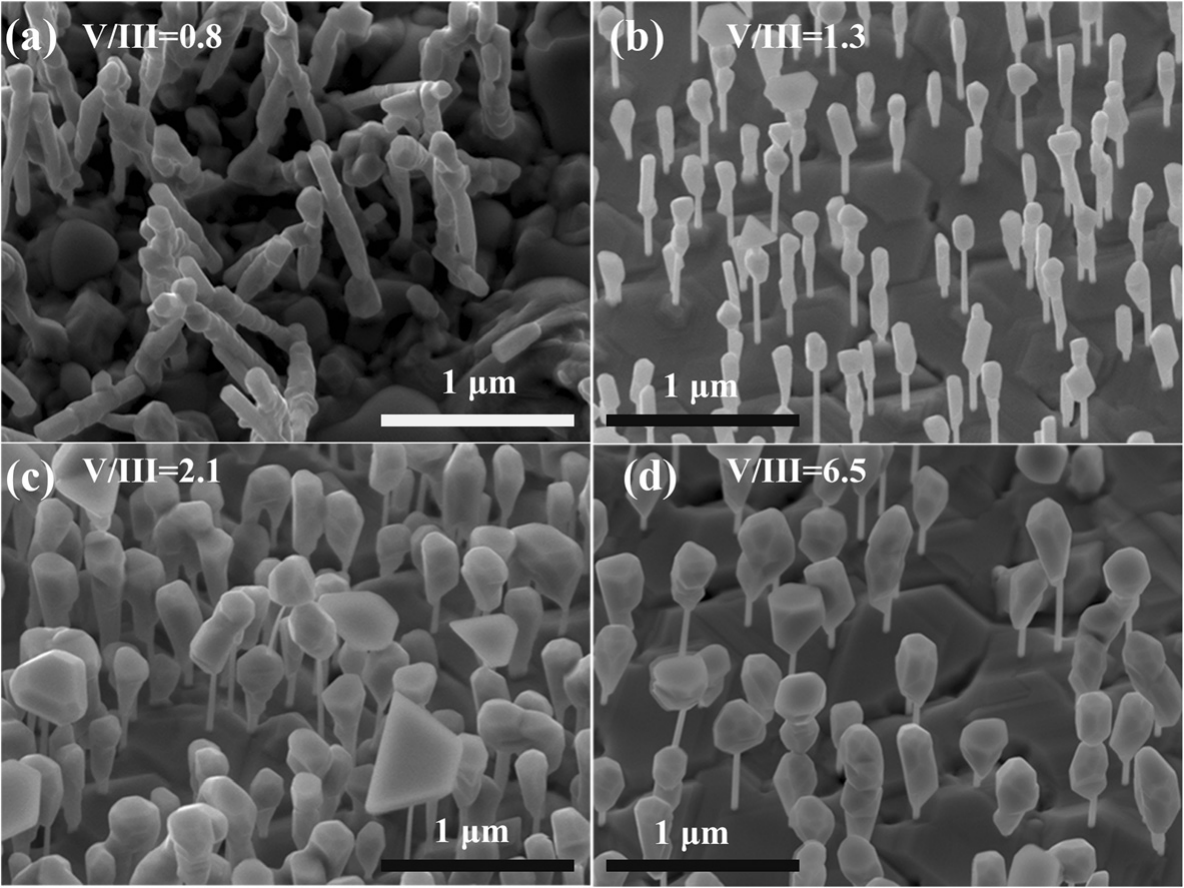
**SEM images of GaAs/GaSb heterostructure NWs grown at different V/III ratios. (a)** V/III = 0.8, **(b)** V/III = 1.3, **(c)** V/III = 2.1, and **(d)** V/III = 6.5, respectively.

Figure 1 shows tilted SEM images of NWs grown at different V/III BEP ratios. When the V/III BEP ratio is 0.8 (Figure 1a), NWs manifest very irregular orientations, and they usually stick together. Meanwhile, the obvious substrate surface irregularity suggests the 2-D thin film growth on the GaAs substrate. Then, when the V/III BEP ratio is increased to 1.3, most NWs grow vertically on the GaAs substrate, as can be observed in Figure 1b, in which most of NWs can be divided into two segments with different diameters. In fact, the upper thicker NW segment has a faceted shape. NWs grown at the V/III BEP ratio of 2.1 (Figure 1c) and 6.5 (Figure 1d) have a basically similar morphology as NWs shown in Figure 1b, but displays a more obvious tendency of lateral growth. The two-segment morphology of GaAs/GaSb NWs grown at higher V/III ratios provides a useful method to distinguish GaAs and GaSb contributions. For the lower thin NW segments, the average diameter for NWs in Figure 1b, c, d can be estimated as 45 nm from which we anticipate that the lower thinner segment should be GaAs NWs, since all GaAs NWs in these three samples were grown under the same conditions. On this basis, the upper thick segment should be caused by the GaSb growth, at least partially. This agrees with the previous report on the GaSb NW growth, in which lateral growth of GaSb NWs can be enhanced by increasing the V/III ratio [30]. Surprisingly, we do not find obvious Au catalyst particles residing on top of GaAs/GaSb core-shell NWs for all four samples.

**Figure 2:**
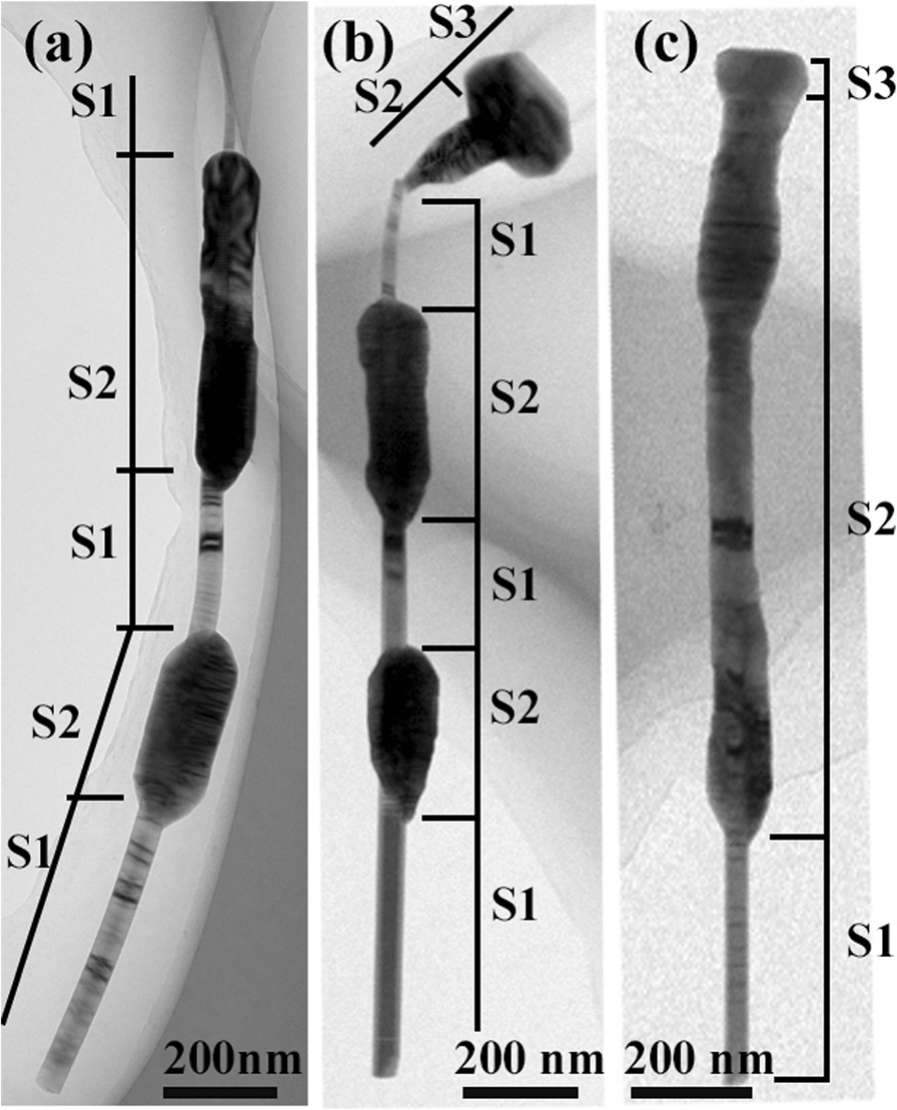
**Three representative morphologies of GaAs/GaSb NWs grown at a V/III ratio of 1.3 (a-c).** Also, three kinds of sections (named as S1, S2, and S3) are identified.

To understand the morphological and structural characteristics of the as-grown NWs, TEM investigation was performed. Figure 2 presents three typical TEM images taken from the NWs grown at a V/III BEP ratio of 1.3. According to their structural and compositional characteristics, three different kinds of regions can be identified in these NWs, which are named as S1, S2, and S3, respectively, as indicated in Figure 2. Specifically, S1 possesses a WZ structure and is composed of pure GaAs; S2 has a highly defected WZ structure and is composed of GaAs core and GaSb shell, while S3 is a pure GaSb bulgy nanoplate with pure ZB structure. Detailed illustrations of S1, S2, and S3 will be given in the following text. Besides, it is also noted that GaSb can either form a continuous or discontinuous shell layer(s) on GaAs cores, which may be attributed to the specific local growth environment of different NWs, as well as the variation of surface energetics.

**Figure 3:**
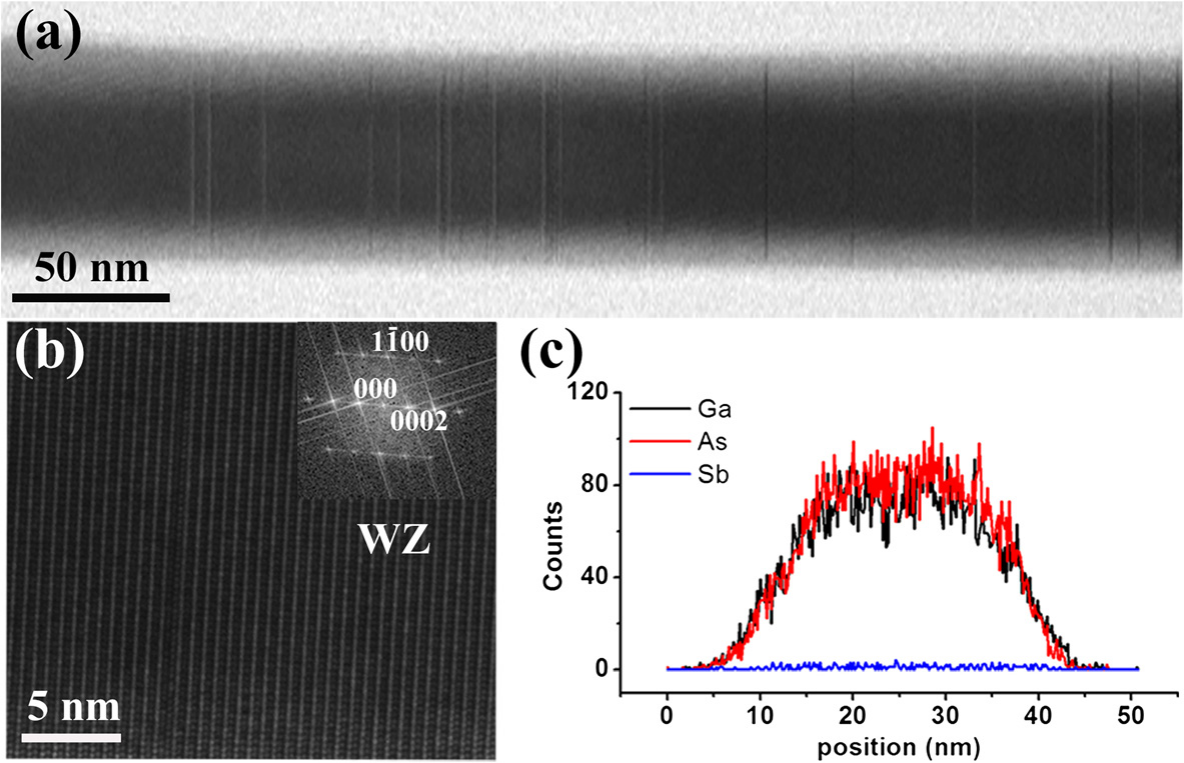
**Structural and compositional details of the S1 segment. (a)** A TEM image of the S1 segment from Figure 2c. **(b)** A high-resolution TEM image clearly showing the WZ structure of the NW segment from (a). **(c)** The typical EDS linescan result along the radial direction, which indicates that the S1 segment is composed of pure GaAs.

Figure 3a shows a high-magnification bright-field TEM image taken from the S1 segment identified in Figure 2c, in which SFs can be occasionally noticed. Figure 3b shows a corresponding high-resolution TEM image, indicating that this section has the WZ crystal structure. Figure 3c shows a representative energy-dispersive X-ray spectroscopy (EDS) linescan of the NW segment shown in Figure 3a and confirms that the WZ segment is indeed pure GaAs. The result is in agreement with our previous study [31].

**Figure 4:**
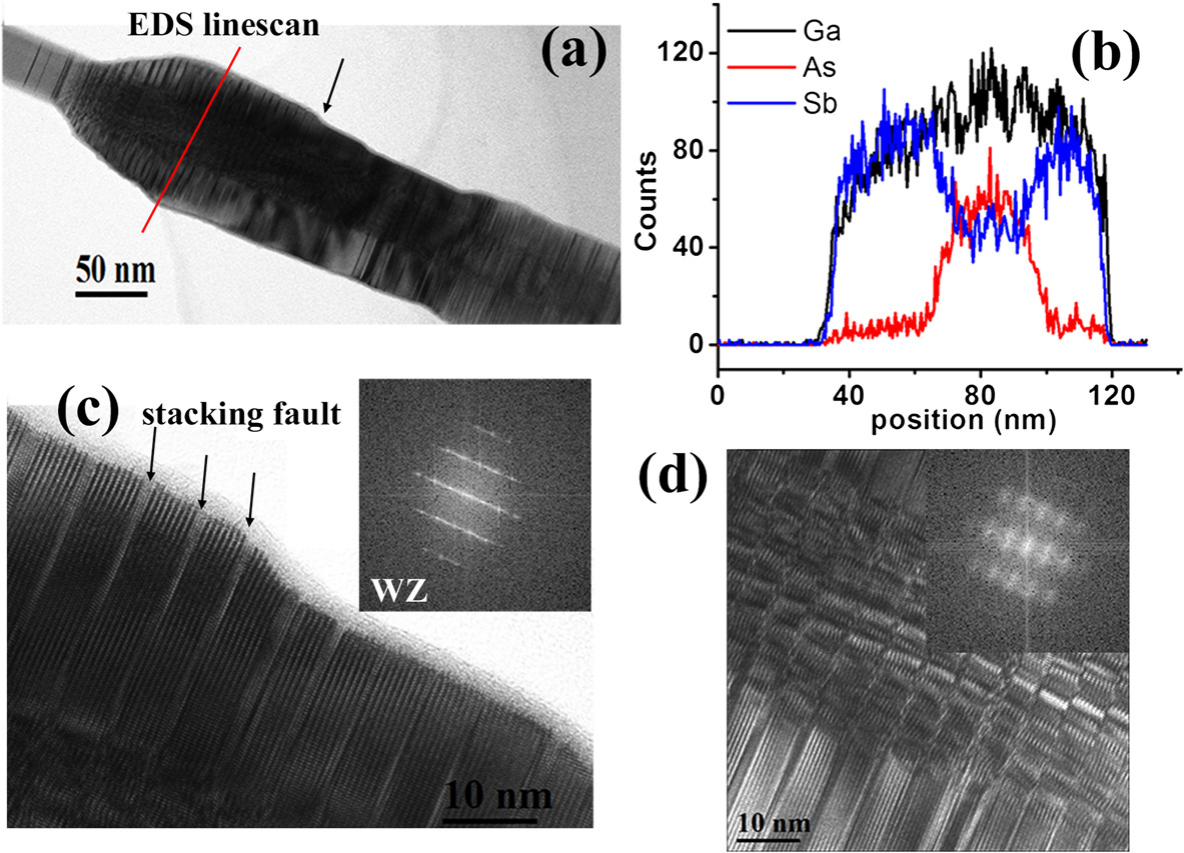
**Structural and compositional details of the S2 segment. (a)** A TEM image of the lower part of the S2 segment from Figure 2c. **(b)** A typical EDS linescan as indicated by a red line in (a), which clearly shows the formation of a GaAs/GaSb core-shell heterostructure. **(c)** A high-resolution TEM image corresponding to the location indicated by a black arrow in (a), which implies a WZ structure of the core-shell NW. **(d)** A typical high-resolution TEM image showing the structural details of the GaAs core. The insets in (c) and (d) are the corresponding FFTs, respectively.

TEM investigations were also performed to understand the crystal structure and chemical composition of the GaAs/GaSb core-shell segment (notated as S2 segment). Figure 4a shows a typical bright-field TEM image of the lowest part of the S2 segment shown in Figure 2c. Figure 4b shows a typical EDS linescan of the NW segment of Figure 4a, which clearly demonstrates the core-shell structure in this nanowire section, with a GaAs core and a GaSb shell. Figure 4c shows a high-resolution TEM image taken from the shell region, in which high density of planar defects can be seen in the WZ-dominant structure. This is due to a large lattice mismatch of 7.5% between the GaAs core and GaSb shell. Figure 4d shows the structural details of the GaAs core and implies that Moire fringes exist in the GaAs core NWs, indicating the strain-induced structural relaxation [16]. Based on the above description, it is found that the GaSb shell layer possesses the WZ structure, which is seldom reported previously.

**Figure 5:**
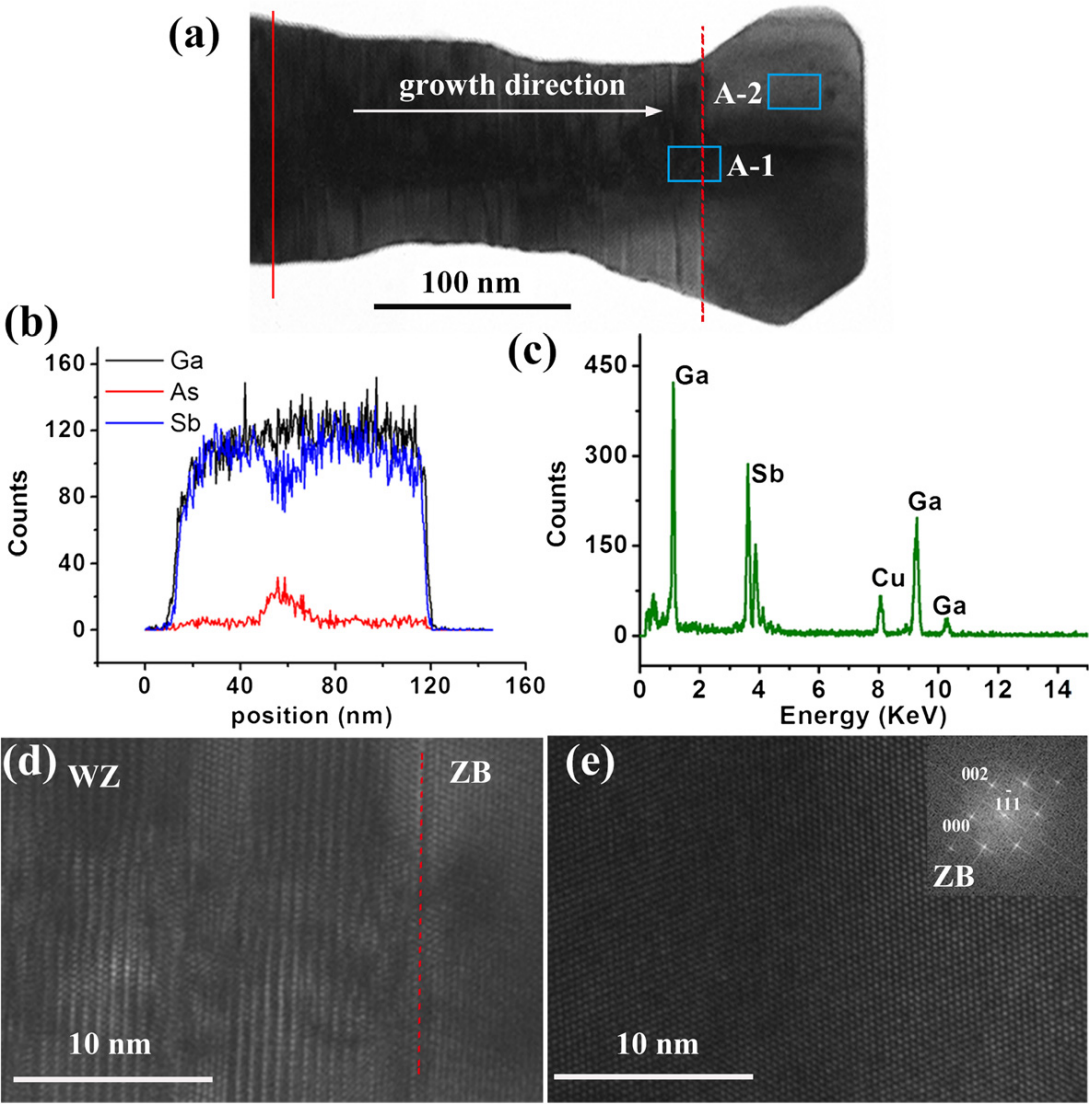
**Structural and compositional details of the S3 segment and its neighboring NW from Figure** 2 **c. (a)** A bright-field TEM image. **(b)** EDS linescan indicated by the red line in (a). **(c)** A representative EDS composition from the S3 segment. **(d-e)** HRTEM images corresponding to the two locations A-1 and A-2 shown in (a).

Figure 5 shows the structural and compositional details of the top bulgy nanoplate and its neighboring segment, with their boundary indicated by a red dashed line in Figure 5a. Figure 5b shows the EDS linescan taken from the red line marked in Figure 5a, from which we find that arsenic signal only covers a radial length of about 10 nm, and its intensity is very small. Compared with Figure 4b in which strong As signal lasts a distance of about 30 nm radially, it can be inferred that GaAs NW should have a tapering shape, and this tapering morphology can also be verified by the TEM image of Figure 2a, b. Similar morphological variation has been well reported [31]. In fact, along the growth direction of the core-shell segment between the red solid line and red dashed line in Figure 5a, arsenic composition decreases quickly, becoming finally undetectable at the boundary position (indicated by the red dashed line). As for the top S3-type nanoplate, Figure 5c presents a typical EDS measurement, which clearly demonstrates its composition of pure GaSb. Figure 5d provides a high-resolution TEM image of the NW segment A-1 shown in Figure 5a, which spans both S2 and S3 segments. A transition from WZ-dominant structure to pure ZB phase is clearly depicted. The HRTEM image of the NW segment A-2 further demonstrates the pure ZB structure of the top GaSb nanoplate (see Figure 5e). Besides, except for the GaSb shell layer grown on GaAs core NW, the pure GaSb top nanoplate also exhibits a tendency of lateral growth, which can be verified more remarkably by the large diameter of the S3 segment in Figure 2b. Our extensive TEM investigations also confirm the formation of core-shell structures in other samples. However, in the previous work reported by de la Mata et al. [32], the axial GaAs/GaSb NW heterostructures have been grown by MBE. But the growth condition adopted by them is quite different from that used in our study. Maybe, the lower growth temperature and smaller V/III ratios used here is more beneficial to the growth of GaSb shell layers. This implies that through careful control of growth parameters, both axial and core-shell GaAs/GaSb NW heterostructures can be obtained in a MBE system.

Besides, careful EDS analyses are also conducted to determine the distribution of Au element on different GaAs/GaSb NWs, since no obvious Au particles can be found on top of these NWs. For most NWs, EDS cannot detect any obvious Au signal, but for some NWs, a much smaller Au particle is found to reside on the middle region of them. This suggests that the Au catalyst particle may diffuse away from the NW top and mainly diffuse into the bulk NW during our GaSb growth; similar results have also been reported in silicon NWs by previous literatures [33-35]. The diffusion behavior of Au atoms has exerted an important influence on the GaSb growth mode.

## Discussion

Based on the experimental results described above, several new phenomena have been discovered. First, instead of the common axial growth, GaSb adopts a lateral growth mode, thus forming shell layers on GaAs core NWs. Second, unlike the widely reported ZB structure for GaSb NWs, GaSb shell layers form a WZ phase. Thirdly, there undergoes a structural transition from WZ to ZB structure when GaSb shell layers change to top bulgy GaSb nanoplates. We believe that these new phenomena may stem from the change of underlying growth mechanism of GaSb segment.

Generally speaking, both the axial growth mode governed by vapor-liquid-solid (VLS) mechanism [36,37] and the lateral growth mode regulated by vapor-solid (VS) mechanism [38,39] can happen in the growth process of III-V NWs. For the axial growth through molecular beam epitaxy, surface diffusion of adatoms acts as the main source for the growth species [40], which is usually driven by the chemical potential difference between the catalyst-NW interfaces. However, the diffusion behavior of Au atoms have directly annihilated the existence of Au catalyst particles residing on top of GaAs/GaSb NWs, this will lead to a seriously weakened driving force for VLS-dominated axial growth, and naturally a preference for VS-dominated lateral growth mode. Moreover, the low growth temperature used here for GaSb growth (only 380°C) in comparison to that from previous reports [24,34] will also contribute to the lateral growth mode dominated by the VS mechanism, because a lower growth temperature will decrease the diffusion ability of reactant atoms, which will lead to the diffusion-limited growth that favors the VS growth mechanism. Therefore, reactant atoms are finally stabilized at nanowire sidewalls, resulting in the nucleation and growth of GaSb shell layers around the GaAs NW cores.

Next, let us discuss why does WZ phase outweigh ZB phase during the growth of GaSb shell layers? In fact, it is commonly found that shell layers usually adopt the same structure as their core NWs for different core-shell NWs, such as InAs/InP [41], GaAs/InAs [42], and GaAs/GaAsSb [43], but a detailed explanation for this phenomenon is seldom discussed. Here, based on the VS growth model [44], the change of Gibbs free energy ∆*G*_VS_ for growing 2D nucleus can be described as:

1 $$ \varDelta {G}_{VS}=-\frac{A\varDelta \mu }{a}+\left(Ph+A\right){\gamma}_{vn}+A{\gamma}_{sn} $$

where *A* is the base (or upper) area of the nucleus, ∆*μ* is the difference of chemical potential for III-V pair between vapor-solid phases, *a* is the area of a III-V pair on the nucleation surface, *P* and *h* are the perimeter and height of the nucleus, respectively, *γ*_vn_ and *γ*_sn_ are the vapor-nucleus interface energy and solid-nucleus interface energy, respectively.

Whether the laterally grown GaSb shell can form a WZ or ZB structure is determined by the relative quantity of ∆*G*_VS_*(WZ) and ∆*G*_VS_*(ZB) (∆G* is the maximum of Gibbs free-energy change). Specifically, the formation of a ZB nucleus on the WZ-GaAs NW sidewall has an additional WZ-ZB phase transition energy (∆γ_P_) or stacking fault energy relative to the formation of WZ nucleus. Meanwhile, the energy of each GaSb pair in WZ phase is higher than that of ZB phase (∆*E* ~ 36 meV from density functional calculations). Therefore, WZ nucleus will become more favorable if ∆γ_P_ > ∆*E*. In other words, the present growth parameters satisfy this condition.

The last remaining question is to understand the growth mechanism of top ZB-structured GaSb bulgy nanoplates. Owing to the lack of Au catalysts, the axial growth of GaSb on the top region of NWs is very similar to the growth of GaSb layers that follows the VS mechanism. In the growth process, the change of Gibbs free energy ∆*G*_VS_ can be described by [44]:

2 $$ \varDelta {G}_{VS}=S{\gamma}_s-\frac{V\varDelta \mu }{b}+\frac{V\varDelta E}{b} $$

where *γ*_s_ is the surface energy of the GaSb nucleus, *S* and *V* are the surface area and volume of the nucleus, respectively, *b* is the volume of each Ga-Sb pair, and ∆*E* is the energy difference of GaSb between WZ and ZB phases. The ∆*E* value is equal to 0 and 36 meV/pair for the growth of ZB and WZ nanowires, respectively. In other words, the growing WZ nucleus has an additional energy than that of the ZB nucleus. Therefore, the growth of ZB GaSb is preferred on the top of nanowires. Owing to the lower growth rate in the top of NWs, the morphology of NWs will be determined by Wulff construction [45,46] in which the facets of NW with low surface energies will be preserved and the facets with high surface energies will disappear, which results in the formation of bulgy GaSb nanoplates. InSb nanocubes [47] and diamond-shaped GaInSb NW segments [48] based on radial overgrowth have also been reported before.

**Figure 6:**
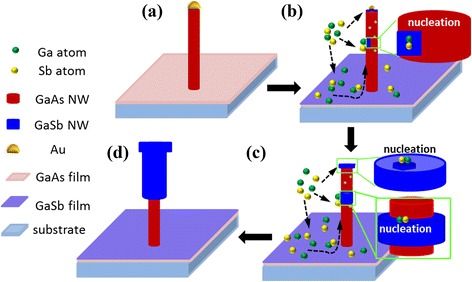
**A schematic illustration for the growth process of GaSb NWs. (a)** The moment of the ending of GaAs growth. **(b)** The initiation stage of GaSb growth, during which Au droplets diffuse away from the NW top, and GaSb growth starts. **(c)** The VS-dominated growth on the NW sidewall and top. **(d)** The final core-shell-structured GaAs/GaSb NWs.

Based on the experimental phenomena and discussions detailed above, a schematic illustration of the growth mode of GaSb NWs is depicted in Figure 6. When GaSb growth starts by the introduction of both Ga and Sb species, the Au catalyst originally residing on the GaAs NW (see Figure 6a) diffuses into the bulk NW, which leads to the seriously weakened driving force for VLS-dominated axial growth. Hence, the VS-dominated lateral growth mode becomes remarkable (see Figure 5b). After the complete disappearance of the Au catalyst, GaSb growth on top of the GaAs NW also adopts a VS mode, just like the shell layer growth on the NW sidewall, as shown in Figure 5c. So, core-shell-structured GaAs/GaSb NWs with a top bulgy nanoplate can be finally obtained.

## Conclusions

To sum up, we have successfully grown GaAs/GaSb core-shell NWs through MBE. Instead of the traditional axial growth commonly reported by many groups, GaSb adopts a lateral growth mode and forms a shell layer on already-grown GaAs NWs and finally has a WZ crystal structure. Meanwhile, a bulgy GaSb nanoplate also appears on top of GaAs/GaSb core-shell NWs and possesses a pure ZB phase. These new phenomena are believed to originate from the lateral growth mode, in contrast to the traditional VLS growth mechanism for axial NW growth. Our study can provide new insight for the growth and understanding of GaSb NW growth, as well as III-V NW growth.

## References

[1] Hu JT, Odom TW, Lieber CM (1999). Chemistry and physics in one dimension: synthesis and properties of nanowires and nanotubes. Accounts Chem Res.

[2] Mourik V, Zuo K, Frolov SM, Plissard SR, Bakkers E, Kouwenhoven LP (2012). Signatures of majorana fermions in hybrid superconductor-semiconductor nanowire devices. Science.

[3] Wernersson LE, Thelander C, Lind E, Samuelson L (2010). III-V nanowires—extending a narrowing road. P IEEE.

[4] Dasgupta NP, Sun JW, Liu C, Brittman S, Andrews SC, Lim J (2014). 25th anniversary article: semiconductor nanowires synthesis, characterization, and applications. Adv Mater.

[5] Kim J, Bahk JH, Hwang J, Kim H, Park H, Kim W (2013). Thermoelectricity in semiconductor nanowires. Phys Status Solidi-Rapid Res Lett.

[6] Hayne M, Young RJ, Smakman EP, Nowozin T, Hodgson P, Garleff JK (2013). The structural, electronic and optical properties of GaSb/GaAs nanostructures for charge-based memory. J Phys D Appl Phys.

[7] Ganjipour B, Ek M, Borg BM, Dick KA, Pistol ME, Wernersson LE (2012). Carrier control and transport modulation in GaSb/InAsSb core/shell nanowires. Appl Phys Lett.

[8] Yang Z, Han N, Wang F, Cheung H-Y, Shi X, Yip S-P (2013). Carbon doping of InSb nanowires for high-performance p-channel field-effect-transistors. Nanoscale.

[9] Kuo C-H, Wu J-M, Lin S-J, Chang W-C (2013). High sensitivity of middle-wavelength infrared photodetectors based on an individual InSb nanowire. Nanoscale Res Lett.

[10] van den Berg JWG, Nadj-Perge S, Pribiag VS, Plissard SR, Bakkers E, Frolov SM (2013). Fast spin-orbit qubit in an indium antimonide nanowire. Phys Rev Lett.

[11] Ganjipour B, Nilsson HA, Borg BM, Wernersson LE, Samuelson L, Xu HQ (2011). GaSb nanowire single-hole transistor. Appl Phys Lett.

[12] Dey AW, Svensson J, Borg BM, Ek M, Lind E, Wernersson LE (2013). GaSb nanowire pFETs for III-V CMOS. 2013 71st Annual Device Research Conference (DRC).

[13] Ganjipour B, Sepehri S, Dey AW, Tizno O, Borg BM, Dick KA (2014). Electrical properties of GaSb/InAsSb core/shell nanowires. Nanotechnology.

[14] Balgos MH, Jaculbia R, Defensor M, Afalla JP, Ibanes JJ, Bailon-Somintac M (2014). Shell to core carrier-transfer in MBE-grown GaAs/AlGaAs core-shell nanowires on Si(100) substrates. J Lumin.

[15] Zhou HL, Hoang TB, Dheeraj DL, van Helvoort ATJ, Liu L, Harmand JC (2009). Wurtzite GaAs/AlGaAs core-shell nanowires grown by molecular beam epitaxy. Nanotechnology.

[16] Salehzadeh O, Kavanagh KL, Watkins SP (2013). Growth and strain relaxation of GaAs and GaP nanowires with GaSb shells. J Appl Phys.

[17] Glas F, Harmand JC, Patriarche G (2007). Why does wurtzite form in nanowires of III-V zinc blende semiconductors?. Phys Rev Lett.

[18] Zhenyu L, Zhi Z, Pingping C, Suixing S, Luchi Y, Chen Z (2014). Bismuth-induced phase control of GaAs nanowires grown by molecular beam epitaxy. Appl Phys Lett.

[19] Mandl B, Dick KA, Kriegner D, Keplinger M, Bauer G, Stangl J (2011). Crystal structure control in Au-free self-seeded InSb wire growth. Nanotechnology.

[20] Gupta N, Song YP, Holloway GW, Sinha U, Haapamaki CM, LaPierre RR (2013). Temperature-dependent electron mobility in InAs nanowires. Nanotechnology.

[21] Tragardh J, Persson AI, Wagner JB, Hessman D, Samuelson L (2007). Measurements of the band gap of wurtzite InAs1-xPx nanowires using photocurrent spectroscopy. J Appl Phys.

[22] Morkotter S, Funk S, Liang M, Doblinger M, Hertenberger S, Treu J (2013). Role of microstructure on optical properties in high-uniformity In1-xGaxAs nanowire arrays: evidence of a wider wurtzite band gap. Phys Rev B.

[23] Kriegner D, Assali S, Belabbes A, Etzelstorfer T, Holy V, Schulli T (2013). Unit cell structure of the wurtzite phase of GaP nanowires: X-ray diffraction studies and density functional theory calculations. Phys Rev B.

[24] Caroff P, Dick KA, Johansson J, Messing ME, Deppert K, Samuelson L (2009). Controlled polytypic and twin-plane superlattices in III-V nanowires. Nat Nanotechnol.

[25] Algra RE, Verheijen MA, Borgstrom MT, Feiner LF, Immink G, van Enckevort WJ (2008). Twinning superlattices in indium phosphide nanowires. Nature.

[26] Bolinsson J, Caroff P, Mandl B, Dick KA (2011). Wurtzite-zincblende superlattices in InAs nanowires using a supply interruption method. Nanotechnology.

[27] Akiyama T, Yamashita T, Nakamura K, Ito T (2010). Band alignment tuning in twin-plane superlattices of semiconductor nanowires. Nano Lett.

[28] Mattias Borg B, Wernersson L-E (2013). Synthesis and properties of antimonide nanowires. Nanotechnology.

[29] Pozuelo M, Zhou H, Lin S, Lipman SA, Goorsky MS, Hicks RF (2011). Self-catalyzed growth of InP/InSb axial nanowire heterostructures. J Cryst Growth.

[30] Jeppsson M, Dick KA, Wagner JB, Caroff P, Deppert K, Samuelson L (2008). GaAs/GaSb nanowire heterostructures grown by MOVPE. J Cryst Growth.

[31] Lu ZY, Chen PP, Liao ZM, Shi SX, Sun Y, Li TX (2013). Impact of growth parameters on the morphology and microstructure of epitaxial GaAs nanowires grown by molecular beam epitaxy. J Alloy Compd.

[32] de la Mata M, Magen C, Caroff P, Arbiol J (2014). Atomic scale strain relaxation in axial semiconductor III-V nanowire heterostructures. Nano Lett.

[33] Hannon JB, Kodambaka S, Ross FM, Tromp RM (2006). The influence of the surface migration of gold on the growth of silicon nanowires. Nature.

[34] Allen JE, Hemesath ER, Perea DE, Lensch-Falk JL, Li ZY, Yin F (2008). High-resolution detection of Au catalyst atoms in Si nanowires. Nat Nanotechnol.

[35] Moutanabbir O, Isheim D, Blumtritt H, Senz S, Pippel E, Seidman DN (2013). Colossal injection of catalyst atoms into silicon nanowires. Nature.

[36] Wagner RS, Ellis WC (1964). Vapor–liquid–solid mechanism of single crystal growth. Appl Phys Lett.

[37] Wacaser BA, Dick KA, Johansson J, Borgstrom MT, Deppert K, Samuelson L (2009). Preferential interface nucleation: an expansion of the VLS growth mechanism for nanowires. Adv Mater.

[38] Lee JS, Brittman S, Yu D, Park H (2008). Vapor–liquid–solid and vapor-solid growth of phase-change Sb2Te3 nanowires and Sb2Te3/GeTe nanowire heterostructures. J Am Chem Soc.

[39] Dimakis E, Lahnemann J, Jahn U, Breuer S, Hilse M, Geelhaar L (2011). Self-assisted nucleation and vapor-solid growth of in as nanowires on bare Si(111). Crystal Growth Design.

[40] Dubrovskii VG, Sibirev NV, Cirlin GE, Soshnikov IP, Chen WH, Larde R (2009). Gibbs-Thomson and diffusion-induced contributions to the growth rate of Si, InP, and GaAs nanowires. Phys Rev B.

[41] Liu XY, Liu PB, Huang H, Chen CX, Jin TN, Zhang YF (2013). Growth and large-scale assembly of InAs/InP core/shell nanowire: effect of shell thickness on electrical characteristics. Nanotechnology.

[42] Rieger T, Schapers T, Grutzmacher D, Lepsa MI (2014). Crystal phase selective growth in GaAs/InAs core-shell nanowires. Crystal Growth Design.

[43] Ghalamestani SG, Munshi AM, Dheeraj DL, Fimland BO, Weman H, Dick KA (2013). Self-catalyzed MBE grown GaAs/GaAsxSb1-x core-shell nanowires in ZB and WZ crystal structures. Nanotechnology.

[44] Markov IV (2006). Crystal growth for beginners: fundamentals of nucleation, crystal growth and epitaxy.

[45] Moll N, Kley A, Pehlke E, Scheffler M (1996). GaAs equilibrium crystal shape from first principles. Phys Rev B.

[46] Wulff GZ (1901). On the question of the rate of growth and dissolution of crystal surfaces. Kristallogr Mineral.

[47] Plissard SR, Slapak DR, Verheijen MA, Hocevar M, Immink GWG, van Weperen I (2012). From InSb nanowires to nanocubes: looking for the sweet spot. Nano Lett.

[48] Ghalamestani SG, Ek M, Ganjipour B, Thelander C, Johansson J, Caroff P (2012). Demonstration of defect-free and composition tunable GaxIn1-xSb nanowires. Nano Lett.

